# Supplementary material for: Tongue Swab Xpert MTB/RIF Ultra Testing for Tuberculosis Using a Revised Consensus Protocol: A multi-country diagnostic accuracy study
Source: medRxiv. 2025 Jul 10:2025.07.08.25330424. Preprint. [Version 1] doi: 10.1101/2025.07.08.25330424 (PMC12265798; doi:10.1101/2025.07.08.25330424)
Supplement: 1 [file NIHPP2025.07.08.25330424V1-supplement-1.pdf]

SUPPLEMENTAL TABLES

Table S1. Tongue swab Xpert Ultra Results by country (N=1168).

	Overall (N=1168)	Philippines (N=295)	South Africa (N=272)	Zambia (N=326)	Nigeria (N=275)
Negative	963 (82.5%)	254 (86.1%)	203 (74.6%)	281 (86.2%)	225 (81.8%)
Non-actionable (error/invalid/no result)	65 (5.6%)	11 (3.7%)	42 (15.4%)	8 (2.5%)	4 (1.5%)
Positive	140 (12.0%)	30 (10.2%)	27 (9.9%)	37 (11.4%)	46 (16.7%)
High	0 (0%)	0 (0%)	0 (0%)	0 (0%)	0 (0%)
Medium	6 (4.3%)	0 (0%)	1 (3.7%)	3 (8.1%)	2 (4.4%)
Low	81 (57.9%)	15 (50.0%)	17 (63.0%)	20 (54.1%)	29 (63.0%)
Very Low	35 (25.0%)	7 (23.3%)	5 (18.5%)	11 (29.7%)	12 (26.1%)
Trace	18 (12.9%)	8 (26.7%)	4 (14.8%)	3 (8.1%)	3 (6.5%)

medRxiv preprint doi: https://doi.org/10.1101/2025.07.08.25330424; this version posted July 10, 2025. The copyright holder for this preprint (which was not certified by peer review) is the author/funder, who has granted medRxiv a license to display the preprint in perpetuity.
It is made available under a CC-BY 4.0 International license .

medRxiv preprint doi: https://doi.org/10.1101/2025.07.08.25330424; this version posted July 10, 2025. The copyright holder for this preprint (which was not certified by peer review) is the author/funder, who has granted medRxiv a license to display the preprint in perpetuity.
It is made available under a CC-BY 4.0 International license .

Figure S1. Non-actionable tongue swab Xpert Ultra results over time, by enrollment site.

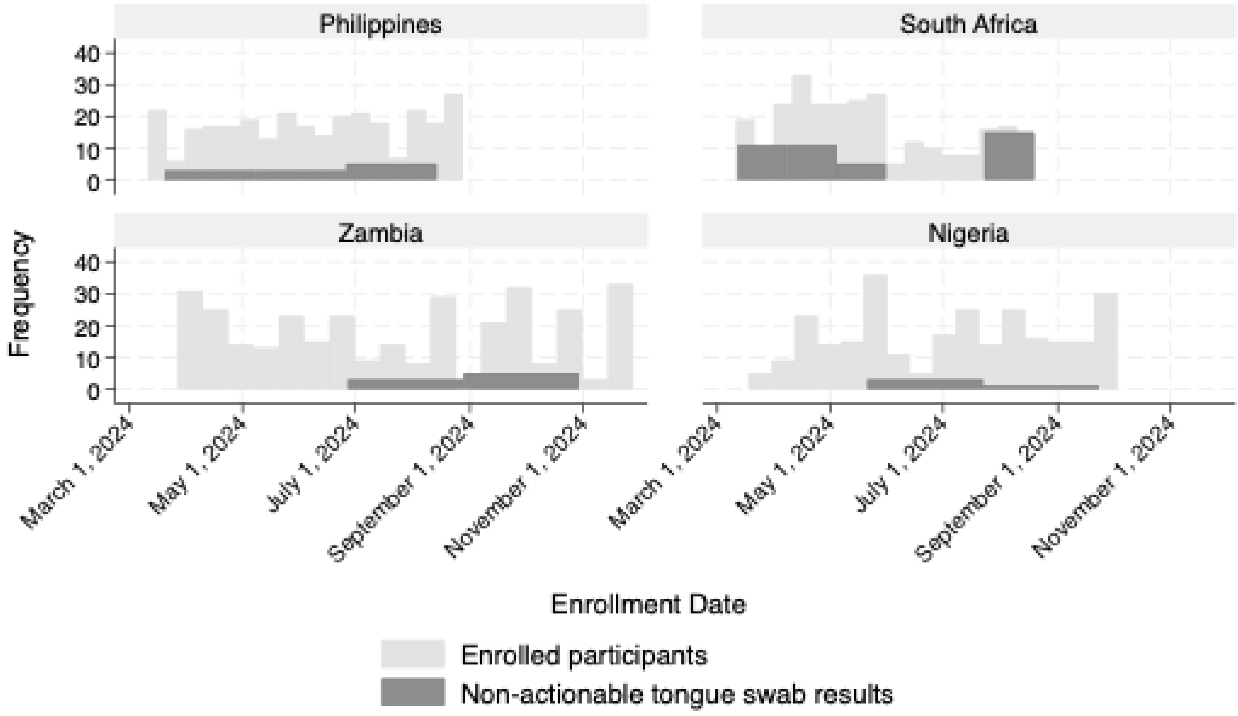

Table S2. Characteristics associated with non-actionable tongue swab Xpert Ultra results (N=1165)[a].

	OR (95% CI)	p-value
Age, years	1.01 (0.99, 1.03)	0.28
Enrollment country		
Nigeria	Ref	
Philippines	2.70 (0.84, 8.67)	0.095
South Africa	11.39 (4.00, 32.45)	<0.001
Zambia	1.59 (0.47, 5.40)	0.46
Sex		
Male	Ref	
Female	1.16 (0.68, 1.96)	0.59
HIV Status		
Negative	Ref	
Positive	1.46 (0.81, 2.62)	0.21

[a] Excludes n=2 with missing HIV status
Abbreviations: CI, confidence interval; OR, odds ratio

Table S3. Diagnostic performance of tongue swab Xpert Ultra compared to sputum Xpert Ultra: Secondary analysis* (N=1012)[a].

	Sensitivity			Specificity		
	% (95%CI) [n/N]	Difference, % (95% CI)	p-value	% (95%CI) [n/N]	Difference, % (95% CI)	p-value
Tongue swab Xpert Ultra	66.7 (59.6, 73.2) [130/195]	-21.0	<0.001	99.6 (98.9, 99.9) [814/817]	0.9	0.065
Sputum Xpert Ultra	87.7 (82.2, 92.0) [171/195]	(-27.4, -14.6)		98.8 (97.8, 99.4) [807/817]	(-0.06, +1.8)	

* Considers sputum Xpert Ultra trace results as positive

[a] Excludes n=80 participants with indeterminate MRS result, n=63 with non-actionable tongue swab Xpert Ultra result and n=13 with non-actionable sputum Xpert Ultra result

Abbreviations: TB, tuberculosis; CI, confidence interval

medRxiv preprint doi: https://doi.org/10.1101/2025.07.08.25330424; this version posted July 10, 2025. The copyright holder for this preprint (which was not certified by peer review) is the author/funder, who has granted medRxiv a license to display the preprint in perpetuity. It is made available under a CC-BY 4.0 International license .
